# The Health and Climate Benefits of Economic Dispatch in China’s Power System

**DOI:** 10.1021/acs.est.2c05663

**Published:** 2023-02-09

**Authors:** Qian Luo, Fernando Garcia-Menendez, Haozhe Yang, Ranjit Deshmukh, Gang He, Jiang Lin, Jeremiah X. Johnson

**Affiliations:** † Department of Civil, Construction, and Environmental Engineering, North Carolina State University, Raleigh, North Carolina 27695, United States; ‡ Bren School of Environmental Science and Management, University of California at Santa Barbara, Santa Barbara, California 93117, United States; § Department of Technology and Society, Stony Brook University, Stony Brook, New York 11794, United States; ∥ Department of Electricity Market and Policy, Lawrence Berkeley National Laboratory, Berkeley, California 94720, United States; ⊥ Department of Agricultural and Resources Economics, University of California at Berkeley, Berkeley, California 94720, United States

**Keywords:** power system in China, air pollution, public health

## Abstract

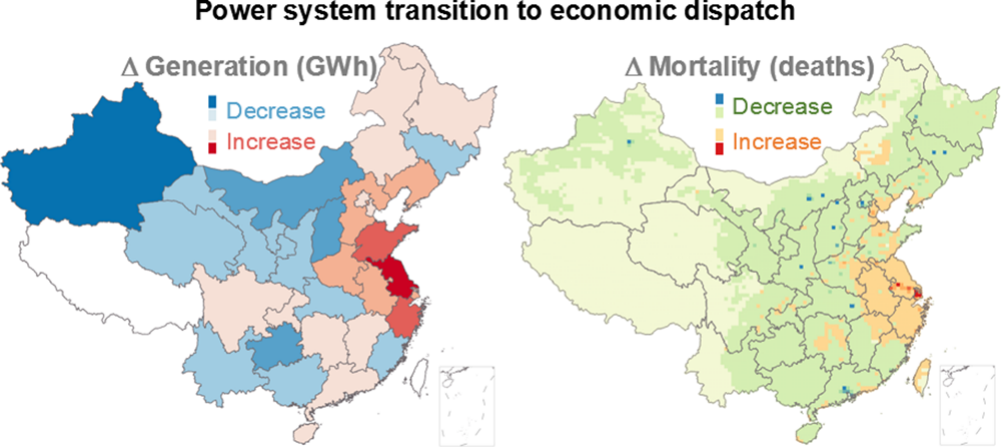

China’s power system is highly regulated and uses an “equal-share” dispatch approach. However, market mechanisms are being introduced to reduce generation costs and improve system reliability. Here, we quantify the climate and human health impacts brought about by this transition, modeling China’s power system operations under economic dispatch. We find that significant reductions in mortality related to air pollution (11%) and CO_2_ emissions (3%) from the power sector can be attained by economic dispatch, relative to the equal-share approach, through more efficient coal-powered generation. Additional health and climate benefits can be achieved by incorporating emission externalities in electricity generation costs. However, the benefits of the transition to economic dispatch will be unevenly distributed across China and may lead to increased health damage in some regions. Our results show the potential of dispatch decision-making in electricity generation to mitigate the negative impacts of power plant emissions with existing facilities in China.

## Abstract 2

By improving the electricity generation efficiency, transitioning to full economic dispatch in China’s power sector would reduce CO_2_ emissions and air pollution-related mortalities. Considering emission externalities in system operations would further mitigate those negative impacts.

## Introduction

China, a country undergoing rapid economic development, has high emissions of air pollutants with negative impacts on the climate and human health.^1−5^ As the world’s largest greenhouse gas emitter, China contributes 30% of global CO_2_-equivalent emissions.^6,7^ Coal combustion is responsible for 80% of the country’s CO_2_ emissions.^8^ Coal is also the dominant energy source in China’s power sector, accounting for 61% and 49% of total electricity generation and installed capacity, respectively, in 2020.^9^ Because of the reliance on coal, electricity generation contributes 40% of the total CO_2_ emissions in China.^10−12^ The power sector is additionally responsible for a large fraction of China’s air pollution,^13,14^ with SO_2_ and NO_*x*_ emissions acting as important precursors to secondary fine particulate matter (PM_2.5_) in the atmosphere and leading to significant negative human health impacts.^15^ Although control measures for power plants have been adopted nationwide, electricity generation still contributes 17% and 19% of the national total SO_2_ and NO_*x*_ emissions, respectively.^16^ Recent studies estimate that 90 000–240 000 annual premature deaths in the country are associated with power plant emissions, representing 10–20% of the mortalities attributable to PM_2.5_ exposure in China.^17−19^

The power sector in China, the world’s largest electricity producing country, is highly regulated, and it uses an “equal-share” dispatch system.^20^ Under this approach, thermal generators of the same class are allocated an equal amount of annual producing hours, regardless of efficiency. In an effort to reduce generation costs and improve system reliability, China has started reforming the power sector by introducing market mechanisms into operations.^21^ In market mechanisms, electricity generation and dispatch is determined by economic dispatch, which optimizes system operations by minimizing electricity generation costs. Several studies have assessed the impacts of adopting economic dispatch in China. Kahrl et al. found that energy efficient dispatch in the Chinese province of Guangxi would not deliver significant energy and cost savings because efficient generators already accounted for most thermal generation in the province.^22^ However, Abhyankar et al. reported a potential 20% reduction in electricity generation costs and a 7% decrease in CO_2_ emissions after adopting economic dispatch in the Southern power grid, a regional grid covering five provinces, due to improved efficiency and reduced hydropower curtailment.^23^ At a national level, Wei et al. showed that economic dispatch across coal-fired power plants can result in benefits due to large variability in the heat rates of coal-fired generators, reducing the power sector’s consumption of coal by 6% and total carbon emissions in China by 3%.^24^ While these studies evaluate the economic impacts or CO_2_ emission reductions associated with economic dispatch, they do not consider the health impacts brought about by this transition.

In this study, we estimate the potential benefits to both climate and human health of economic dispatch adoption by China’s power system. We also evaluate the benefits of explicitly considering emission externalities in electricity generation and dispatch operations. To do this, we use a power system optimization model to simulate hourly electrical grid operations in China under unit commitment and economic dispatch for 8760 h in a year, minimizing operational costs. We then evaluate the changes in operations when emission externalities are monetized and internalized. Finally, a reduced-form air quality model is used to simulate air pollutant concentrations and quantify negative health impacts associated with power plant emissions under different power system operation scenarios. We find that the transition from equal-share to economic dispatch in China would improve the power system’s efficiency and yield benefits to both climate and human health. These benefits would be realized even though a larger fraction of electricity would be generated from coal combustion due to the high price of natural gas. We also demonstrate that incorporating emission externalities into operational decisions would further reduce CO_2_ emissions and health impacts of power plant emissions, with the benefits varying across different regions in China.

## Methods

### Power System Operations in China

Currently, China’s power system largely operates on the basis of an equal-share dispatch approach. To represent coal- and gas-fired generation in China at the plant level in a power system model, we first obtained annual generation by energy resource in each province from the China Statistical Yearbook 2020.^25^ We allocated the generation to individual power plants by assuming that plants using similar fuels are run for approximately the same number of hours in a year. To simulate power system operations under unit commitment and economic dispatch, we obtained power grid information from the SWITCH-China model and used the GridPath model to determine hourly coal-fired generation at the unit level and generation from other energy sources at the plant level for the year 2020.^26−29^ Approximately 5000 unique units are represented in the power system model, about 3000 of which are coal-fired generators (54% of total installed capacity, Figure S1). Information about the location, heat rate, and capacity of the coal-fired power plants was obtained from the Global Coal Power Plant Tracker for the year 2020.^30^Figures S2 and S3 show the distribution of unit-level coal heat rates by region and capacity, respectively. Capacities and heat rates for other existing power plants are from He et al.^27^ GridPath is a versatile platform to conduct analyses related to power system planning. We used the production-cost approach in this work, which includes detailed operational constraints and minimizes system costs for a specified power system. GridPath optimizes power system operations by using mixed integer linear programming and captures important operational constraints, including constraints on the balance between load and generation, generator up-/downtime, operational range, ramping, and transmission limits. GridPath v10.0 is used in this study, and detailed documentation for GridPath is available at: https://github.com/blue-marble/gridpath.^29^ In the power system optimization model, every province in mainland China is treated as a load zone, except for Inner Mongolia, which is divided into West Inner Mongolia and East Mongolia. Tibet is not included in the system due to the lack of data. We assume there are no transmission limits within each balancing zone. Hourly load data are from Abhyankar et al. and capture diurnal variation in both working days and holidays.^31^ Hourly wind and solar capacity factors in each load zone are also obtained from Abhyankar et al.^31^ and calibrated against renewable capacity factors in 2020.^25^ Monthly average hydropower capacity factors are from He et al.^27^ To calibrate wind and solar profile inputs, we obtained their annual capacity factors at each province from the China Statistical Yearbook 2020 and then modified the hourly capacity factor by applying a ratio between the recorded annual capacity factor in 2020 and the annual capacity factor estimated by Abhyankar et al. at each load zone without changing the profile shape.^25,31^ Although the historical renewable capacity factor is reported after curtailment, national curtailment of wind and solar was only 3% and 2% in 2020.^32,33^ Additionally, during the simulated year, the model only considers existing transmission lines, and no new lines were added. Therefore, we assumed that curtailment will remain at the same level after economic dispatch was adopted. Under the externality internalization scenarios, monetized emissions externalities were treated as variable costs of electricity generation and were incorporated into power plant dispatch.

### Air Pollution and Health Impacts

A reduced-form air quality model, InMAP-China,^34^ was used to simulate the annual-average PM_2.5_ concentration associated with SO_2_, NO_*x*_, and PM_2.5_ emissions from the power sector in China. InMAP uses chemistry derived from a state-of-the-science comprehensive regional-scale air quality model (WRF-Chem) and has been shown to perform satisfactorily in simulating PM_2.5_ concentrations over China.^34,35^ A 36-km horizontal resolution was used for the simulations conducted in this study. A log–linear concentration–response function was used to estimate annual premature deaths associated with SO_2_, NO_*x*_, and PM_2.5_ emissions from power plants, and we used a mortality hazard ratio for long-term exposure to PM_2.5_ derived from a large cohort study of Chinese population.^36^ To assess the power sector’s air quality and human health impacts, we first estimated emissions from each coal- and gas-fired power plant using unit-level annual generation and the emission rates. Emission rates varied by location, generation capacity, and the build year of each generator.^37^ We then carried out two InMAP-China simulations of PM_2.5_ concentrations and associated mortality in China: (1) a simulation excluding power sector emissions and (2) a simulation including the emissions estimated for coal- and gas-fired power plants. PM_2.5_ pollution and health impacts attributable to power plant emissions were estimated as the differences between concentrations and mortality predicted by the simulations.

### Marginal Health Damage Costs

In contrast to a carbon tax, marginal health damage costs can vary significantly by location due to differences in meteorology and population density. Here, we estimated the marginal health damage of SO_2_, NO_*x*_, and PM_2.5_ emissions from coal-fired power plants in each province, as coal combustion was responsible for the majority of the adverse human health impacts associated with power plant emissions.^17,19^ We conducted a set of InMAP-China simulations for each load zone, once with the model’s original pollutant emission estimates and again with 10% reductions to power plant SO_2_, NO_*x*_, or PM_2.5_ emissions within the load zone.^34^ The value of statistical life (VSL) is based on an individual’s willingness to pay to reduce their risk of death.^38−40^ This value is commonly used to monetize health damage caused by air pollution and conduct cost-benefit analyses.^41−43^ The decrease in predicted mortality (Δ*M*) caused by the emission reduction (Δ*E*) is used along with the VSL estimate selected to calculate marginal health damage costs as
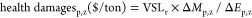
where p is the pollutant considered, z is the load zone selected, and r is the regional VSL if a national value is not used.

We repeated the process for each of the three air pollutants and 31 balancing zones considered to estimate spatially varying marginal health damage costs across China (Figures 3 and S7).

## Results and Discussion

### Power Plant Operations under Economic Dispatch

**Figure 1:**
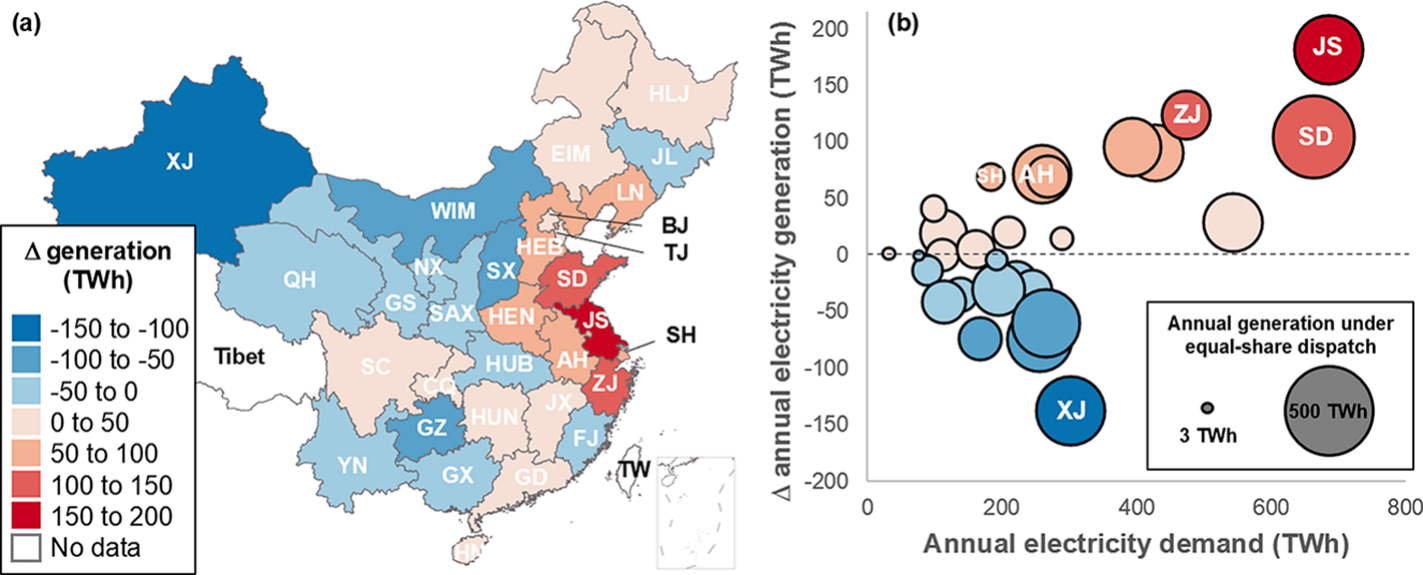
Impacts of economic dispatch on electricity generation in China (2020). (a) Change in annual electricity generation by load zone under economic-dispatch, relative to equal-share dispatch. (b) Regional annual electricity demands and generation changes brought about by economic dispatch. Anhui (AH), Jiangsu (JS), Shandong (SD), Shanghai (SH), Xinjiang (XJ), and Zhejiang (ZJ) provinces are labeled. Abbreviations for each load zone are listed in Table S2.

On the basis of our simulations, transitioning power system operations in China from equal-share to economic dispatch would lead to more electricity generated from coal (356 TWh, a 7.5% increase) and less generated from natural gas due to the low price of coal relative to that of natural gas (Table 1). However, because electricity is produced by more efficient coal-fired power plants, with the average heat rate dropping from 9.65 to 9.12 MMBtu/MWh, total CO_2_ emissions from the power system decrease by 3% under economic dispatch. Under equal-share dispatch, electricity generation in low-load zones (e.g., Xinjiang) usually exceeds demand, and electricity is exported to regions with higher loads. Although long-distance electricity transmission can be expensive, it is not considered in the equal-share dispatch decision-making process. However, under economic dispatch, which considers transmission costs in dispatch decision-making, electricity transmission across load zones is reduced. Therefore, we find that generation in low-load zones drops significantly, and regions with high electricity demand, mostly located in Eastern China (e.g., Jiangsu and Shandong), tend to generate more electricity when transitioning from equal-share to economic dispatch, even though fuel costs are generally lower in western regions (Figure 1). As transmission cost data are limited in China, we test the model’s response to different transmission cost assumptions and find that the distribution of generation would be similar unless transmission costs are close to zero.

**Table 1:** Fossil Fuel-Fired Electricity Generation in China under Equal-Share and Economic Dispatch in 2020

	equal-share	economic dispatch
coal generation (TWh)	4716	5072
natural gas generation (TWh)	435	10
power sector CO_2_ emissions (million tons)	4814	4680
average heat rate of coal generation (MMBtu/MWh)	9.65	9.12

**Figure 2:**
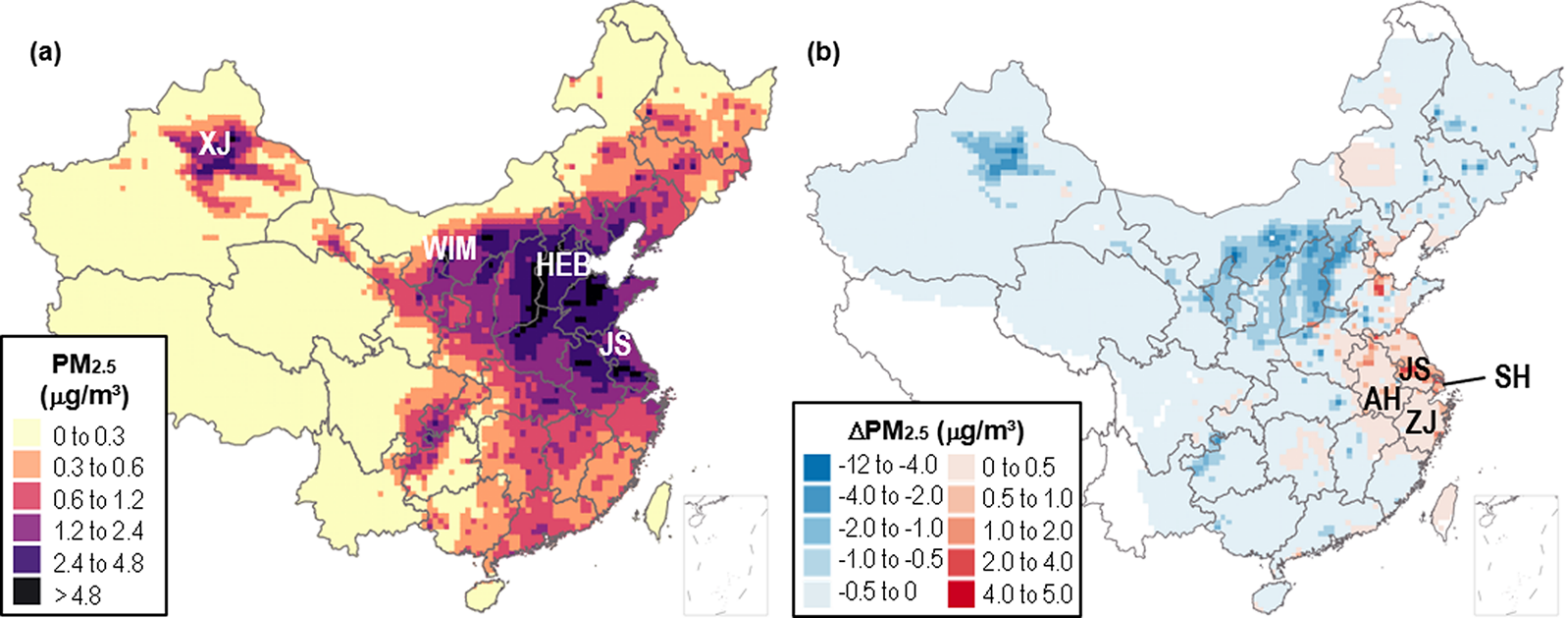
Impacts of economic dispatch on PM_2.5_ pollution in China (2020). (a) Annual-average PM_2.5_ concentrations attributed to power sector emissions in China. (b) Change in annual-average PM_2.5_ concentration under economic dispatch, relative to equal-share. Anhui (AH), Hebei (HEB), Jiangsu (JS), Shanghai (SH), Xinjiang (XJ), West Inner Mongolia (WIM), and Zhejiang (ZJ) provinces are labeled on the map.

In addition to reduced CO_2_ emissions, we also find that total SO_2_, NO_*x*_, and PM_2.5_ emissions from power plants in China would decrease by 21%, 13%, and 44%, respectively, under economic dispatch. Higher energy efficiency in coal generation and stricter air pollution regulations for power plants in eastern regions both contribute to the estimated reduction in emissions. As a result of these lower emissions, atmospheric PM_2.5_ concentrations associated with power generation will drop across most regions in China. In a few eastern provinces (e.g., Anhui, Jiangsu, Shanghai, and Zhejiang), PM_2.5_ concentrations may increase due to greater coal-fired electricity generation (Figures 1 and 2). Economic dispatch can lead to improved air quality in many areas experiencing high PM_2.5_ levels (e.g., Xinjiang, West Inner Mongolia, and Hebei) but also increased PM_2.5_ concentrations in locations where they are relatively high (e.g., Jiangsu) (Figure 2). Although more electricity would be generated in regions with high population density after transitioning from equal-share to economic dispatch, PM_2.5_ concentration reductions across China would yield significant health benefits overall. We estimate that the transition could prevent 8340 premature deaths annually, an 11% reduction in the mortality attributable to the power sector under equal-share dispatch. Similar to the heterogeneous impacts on PM_2.5_ concentrations across regions, not every province would experience human health benefits from the transition. For example, due to increased coal-fired generation and dense population, total annual mortalities associated with power plant emissions in Anhui, Shanghai, Jiangsu, and Zhejiang, located in Eastern China, are projected to increase by 3630 deaths with economic dispatch. Public health across load zones in the South Central and North China would benefit most from economic dispatch. Regions with greater energy demand tend to have lower predicted health benefits due to reduced electricity imports.

### Emission Externalities across Regions and Power Plants

To further explore the potential benefits of emission reductions induced by economic dispatch in China’s power sector, we considered the costs of negative externalities from emissions associated with climate change and human health. The social cost of carbon (SCC) is used to represent climate change externalities and applied to CO_2_ emissions from coal and gas-fired power plants. Here, we used a SCC value specifically estimated for China, $25 per ton of CO_2_ emitted,^44^ and a mean value from 58 studies, $55 per ton of CO_2_ emitted,^45^ yielding two carbon pricing scenarios: CP25 and CP55. The costs of externalities from emissions at each power plant are estimated as a function of its emission rates. Thus, climate externality costs vary across power plants but do not depend on load zone.

**Figure 3:**
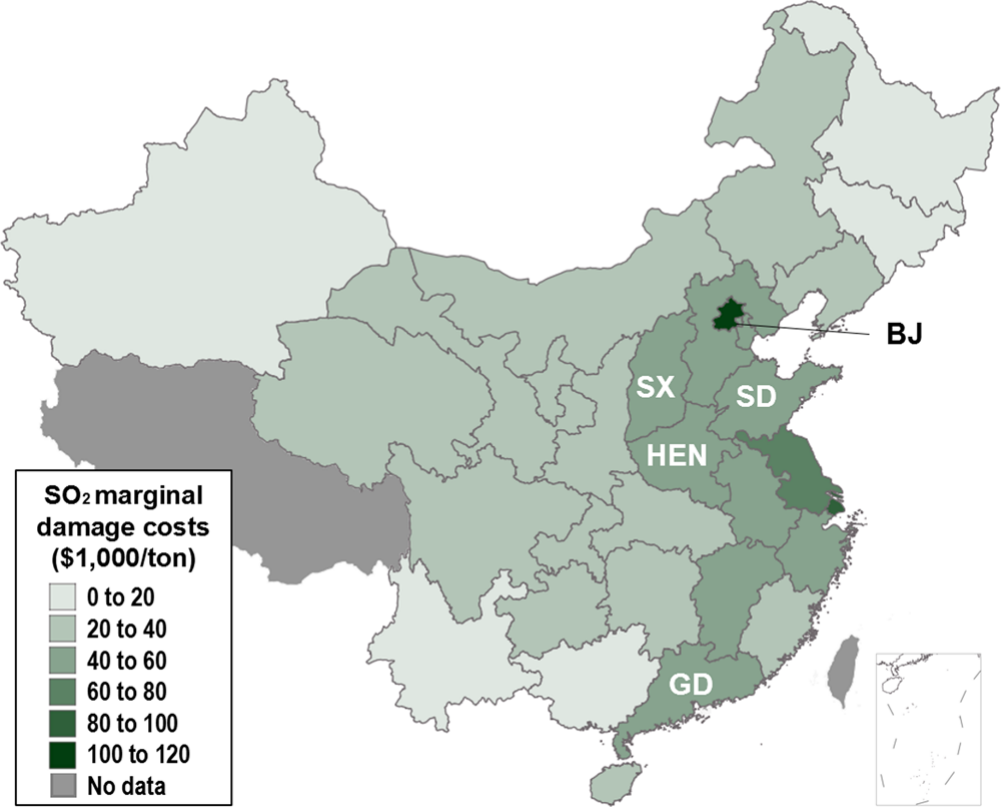
Average marginal health damage costs of SO_2_ power plant emissions estimated for each Chinese province. Estimates are based on a value of statistic life of $1.2 million. Beijing (BJ), Henan (HEN), Guangdong (GD), Shandong (SD), and Shanxi (SX) provinces are labeled.

**Figure 4:**
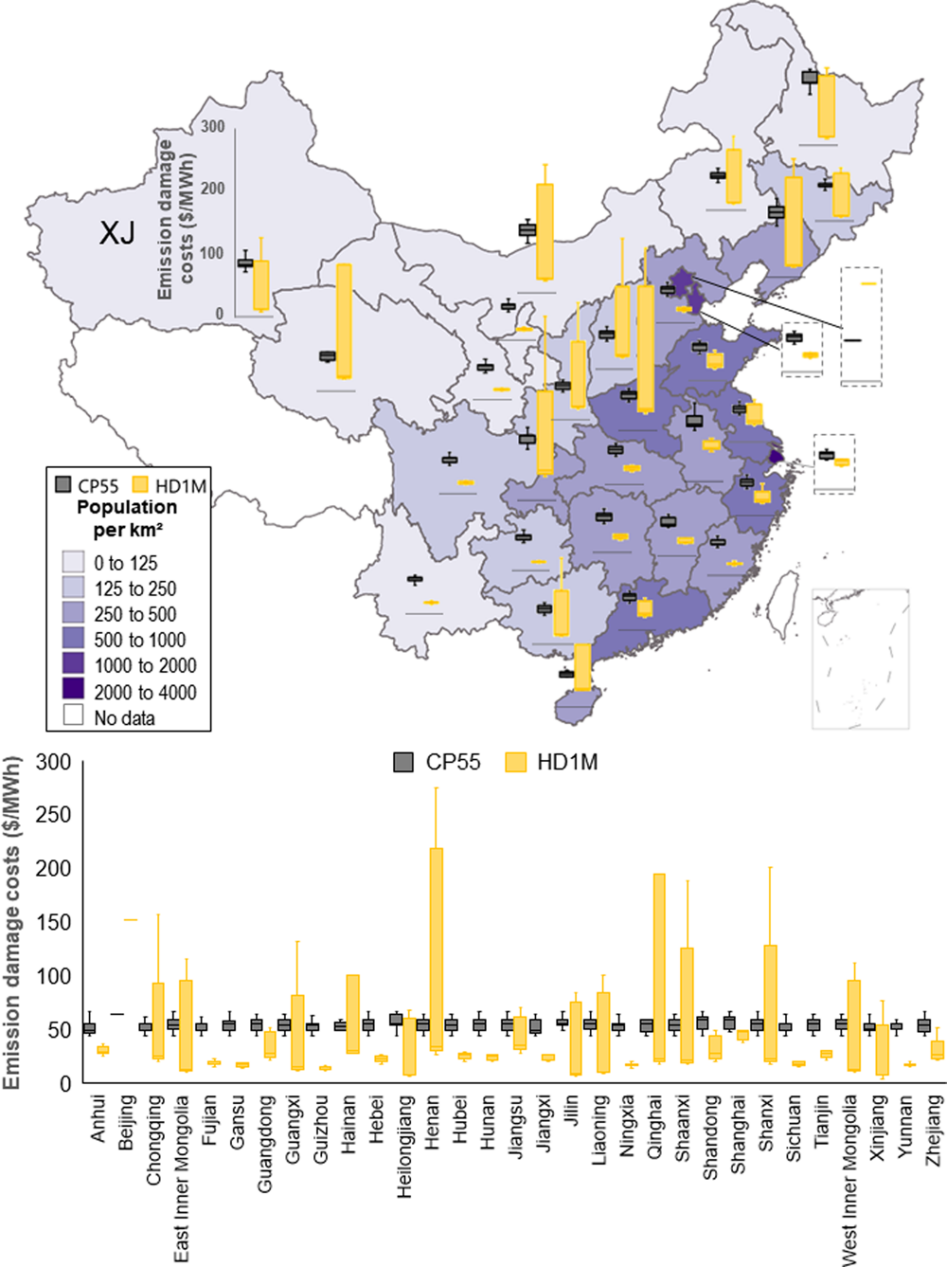
Variability in negative externalities of electricity generation ($ per MWh) within and across load zones. Box plots show the distribution of monetized unit-level emission externalities related to negative climate change and human health impacts for all coal-fired power plants within each Chinese province, based on a carbon emissions price of $55/ton CO_2_ (CP55) and a VSL of $1.2 M/death (HD1M). Box boundaries show the interquartile range, and lines indicate medians. The scale of the box plots is shown in the map for Xinjiang (XJ). Province population densities are also shown.

To consider the human health impacts of air pollution from power plants, we calculated province-level average marginal health damage costs for coal-fired power plants only, which account for most mortalities caused by power sector emissions.^17,19^ Specifically, we quantified the negative impacts of PM_2.5_ on premature mortality, which is responsible for the majority of the human health burden of air pollution.^1,46,47^ We then developed three scenarios based on three values of a statistical life (VSL) to monetize health damage: a national value for China of $1.2 M per death (HD1M),^40^ a value used by U.S. Environmental Protection Agency of $9.5 M per death (HD9M),^38^ and a set of regional values estimated for six cities in China ranging from $0.6 M to $1.0 M per death (HD6cities) .^39^ Because of high population density and weather that often favor the formation of secondary PM_2.5_, estimates of marginal emission damages are generally higher in eastern regions of China, with the largest estimated in Beijing (Figure 3). Marginal costs of NO_*x*_ and PM_2.5_ emissions have spatial distributions similar to those of SO_2_ (shown in Figure S7). Each power plant has different emission rates of SO_2_, NO_*x*_, and PM_2.5_, leading to high variability in the health damage costs ($ per MWh) both within and across load zones (Figure 4). Health damage costs of electricity generation tend to be high in regions with high marginal damages per ton of air pollutant emitted, such as Henan and Shanxi. However, provinces, such as Shandong and Guangdong, where the marginal health damage costs of emissions are relatively high, have low health damage costs per MWh of electricity generated due to low pollutant emission rates under stricter emissions controls in these regions.^37^ As compared to health damage from SO_2_, NO_*x*_, and PM_2.5_, climate externality costs vary less across regions and power plants. Still, under the CP55 scenario, the average climate damage costs across all generators are slightly higher than the average health damage costs based on the HD1M scenario.

### Potential Benefits of Internalizing Emission Externalities

**Figure 5:**
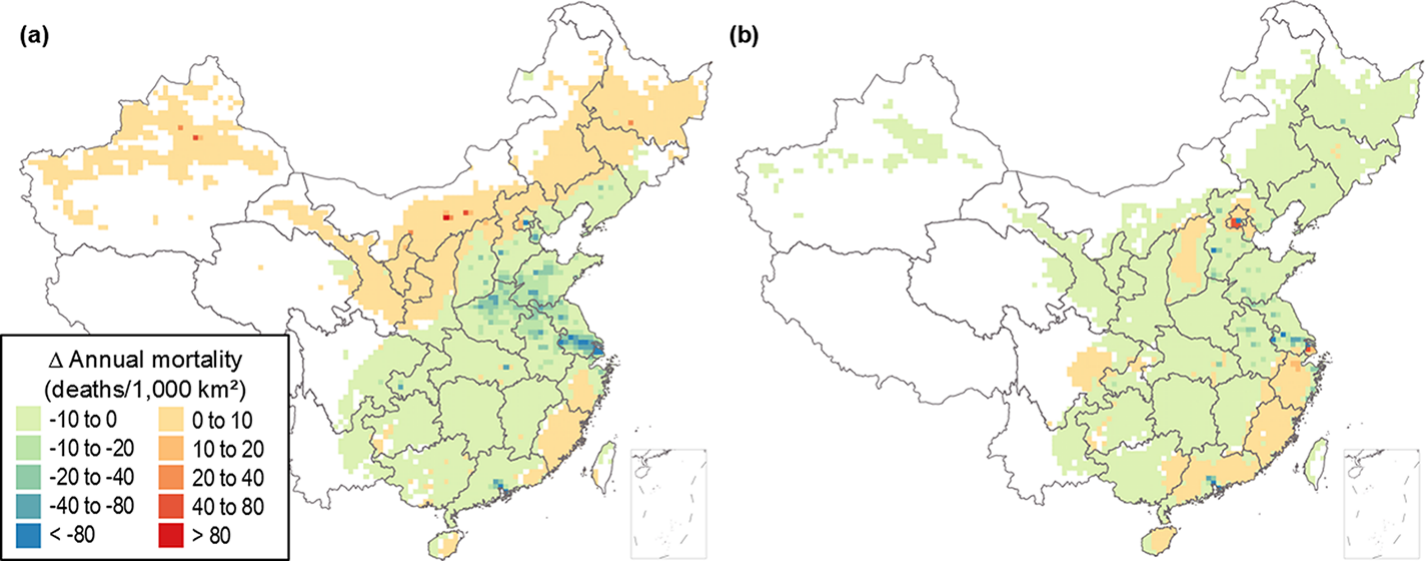
Health impacts of internalizing negative climate and health externalities into power sector operations in China. Change in annual premature deaths associated with PM_2.5_ pollution from the power sector under (a) health damage costs internalization (HD1M) and (b) climate pricing (CP55) scenarios, relative to economic dispatch operations.

**Figure 6:**
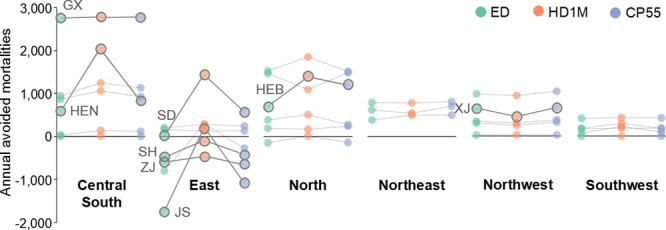
Regional impacts of incorporating health and climate externalities into power system operations. Avoided mortalities associated with power sector emissions in each province are relative to equal-share operations, under economic dispatch (ED), economic dispatch with health damage internalization (HD1M), and economic dispatch with climate impact internalization (CP55) scenarios. Guangxi (GX), Henan (HEN), Hebei (HEB), Jiangsu (JS), Shandong (SD), Shanghai (SH), Xinjiang (XJ), and Zhejiang (ZJ) provinces are labeled.

To explore the effects of health and climate costs internalization into dispatch decision-making in China’s power sector, we incorporate health damage and CO_2_ prices into the power system optimization model as variable electricity generation costs. As compared to economic dispatch solely based on minimizing electricity generation and transmission costs, carbon pricing (CP55) and health damage internalization (HD1M) reduce CO_2_ emissions both by an additional 5%, and premature mortality associated with air pollution from power plants by 5% and 12%, respectively (Table S5). A lower $25/ton tax on CO_2_ emissions (CP25) does not substantially reduce CO_2_ emissions beyond economic dispatch (−1.5%) due to the high price of natural gas, but does result in a 3.7% reduction in premature mortalities, mainly due to the switch in generation among coal-fired power plants. Under the CP55 and HD1M scenarios, adverse health impacts are mitigated by shifting electricity generation to natural gas and decreasing pollutant emissions near population centers. The positive impacts of externality internalization on human health would mostly occur in eastern China, where population and health damage of electricity generation are both high (Figure 5). Health damage internalization generally favors human health in the regions that would already benefit significantly from economic dispatch (e.g., Henan and Hebei, Figure 6). In contrast, some regions with lower population would incur larger adverse health impacts as compared to the economic dispatch scenario that only considers electricity generation and transmission costs (e.g., Xinjiang in Northwest China, Figure 6). However, relative to the equal-share dispatch approach, internalizing health damage negatively impacts human health in only two load zones (Shanghai and Zhejiang in the East, Figure 6). Alternatively, because the CO_2_ prices do not differ across load zones, the mortality associated with power plant emissions is lower in most regions under the CP55 scenario as compared to economic dispatch operations. Mortalities increase in a few areas due to increased use of natural gas. In some regions, Northeast, Southwest, and Northwest China, incorporating climate change externalities into power system operations would not significantly influence the health impacts of air pollution from the power sector beyond the effects of economic dispatch.

**Figure 7:**
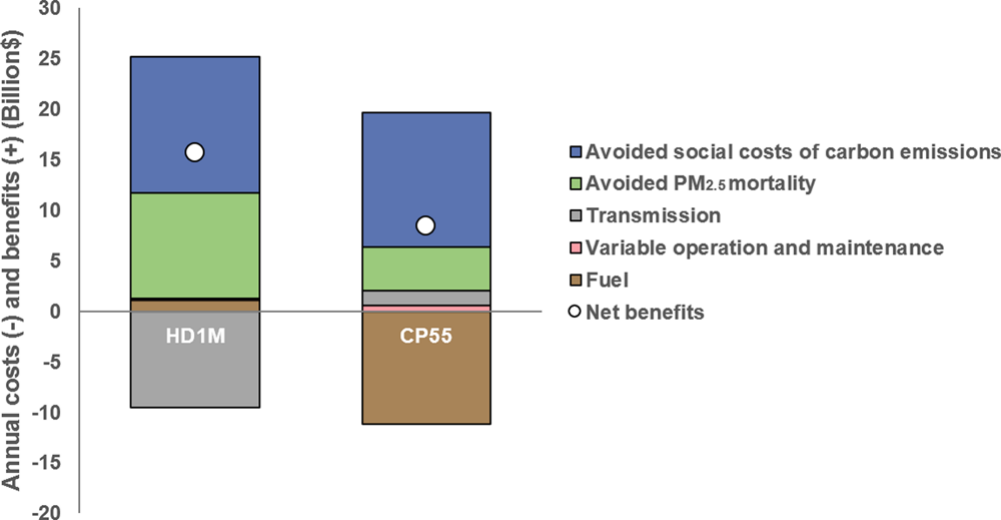
Annual benefits of emissions externalities internalization in power system operations. Additional costs and benefits under health damage internalization (HD1M) and carbon pricing (CP55) scenarios shown are relative to economic dispatch system operations and include fuel, transmission, operation and maintenance, air pollution health impacts, and carbon emissions. All amounts are reported in 2022 USD.

Relative to economic dispatch, which represents operations at their lowest electricity generation and transmission cost, power system cost estimates are higher under the CP55 and HD1M scenarios (Figure 7). System-wide fuel costs rise under the CP55 scenario due to the increased use of natural gas. Transmission costs increase significantly under the HD1M scenario as more electricity is exported from load zones with lower health damage costs to offset generation in regions in which they are higher. Because of the lack of variability in the price of CO_2_ emissions or significant shifts in electricity generation across load zones, carbon pricing mainly reduces emission through increased use of lower-emitting generators (including coal- and gas-fired units) within each load zone. Still, when considering benefits to climate and human health, both the HD1M and the CP55 scenarios have large net benefits ($8–16 billion USD annually) relative to the economic dispatch approach.

## Discussion

The power sector’s transition to economic dispatch operations in China will significantly affect the environment and human health. We project that this transition will bring about large near-term benefits to human health and modest benefits to climate with existing facilities. By generating more electricity from coal-fired power plants, albeit at higher average efficiency, economic dispatch can reduce adverse health impacts of power plant emissions by 11% and CO_2_ emissions by 3%. This would represent a significant shift in the power sector. With the implementation of strict emission controls, the average removal efficiency of SO_2_, NO_*x*_, and PM_2.5_ emissions across coal-fired power plants in China has reached 89%, 62%, and 97%, respectively.^19^ As a result, mortality associated with air pollution attributable to power plant emissions in China has also dropped significantly.^19^ Economic dispatch can further reduce the power sector’s human health burden by reducing generation from the highest-polluting plants. As for carbon emissions, from 1978 to 2018, the carbon intensity of energy consumed in China dropped by only 13%.^48^ Economic dispatch can reduce CO_2_ emissions by 3% through improved efficiency without any new infrastructure or emission control investments. Although market mechanisms have been introduced to power system operations in China, only 30% of electricity is traded.^9^ This study quantifies the range of potential health and climate benefits from adopting economic dispatch relative to a baseline of full equal-share operations. Although the emissions inventory is developed on the basis of full equal-share dispatch, our estimates of total mortality caused by power plant emissions (76 000 annual deaths) are close to those estimated using historical emissions (93 000 annual deaths in 2015, Wu et al.^19^).

To investigate opportunities to reduce the negative impacts of electricity generation further, we incorporated emission externalities into power system operations, which has been shown to yield climate and health benefits in previous studies.^23,49−51^ However, similar to the findings in Lin et al.,^21^ we find that low carbon prices ($25 per ton of CO_2_ emissions) would not appreciably reduce carbon emissions from the power sector in China, as generation with natural gas remains more expensive than that with coal. At a price of $55 per ton, electricity generation shifts from coal to gas, resulting in both climate and human health benefits. To quantify health damage, we explored the sensitivity of health damage internalization results to monetization of health benefits by testing two additional sets of VSL estimates: regional values estimated for six Chinese cities and the value used by the U.S. Environmental Protection Agency for mortality risk valuation ($9.5 M per death).^38−40^ The results obtained by applying regional VSL estimates were similar to those based on a national value ($1.2 M per death). This is due to limited differences in the VSL across regions, which leads to the variability in the health damage costs being driven by population density and meteorology, and the average regional VSL being close to the national value used in the HD1M scenario. When the U.S. Environmental Protection Agency’s much higher VSL estimate is applied, fewer premature deaths associated with power plant emissions are projected. However, the difference in benefits is not as large as might be expected from the large difference in VSL estimates due to the limited capacity of existing natural gas-fired power plants; under the health damage internalization scenario, all gas-fired plants operated at full capacity year-round to minimize health damage costs. Internalization of health damage costs into power system operations can influence the distribution of air pollution-related health impacts. Regions with lower population are negatively affected by the internalization, relative to economic dispatch based on electricity generation and transmission costs alone, and using a higher VSL exacerbates these adverse effects. Further, regions in China with higher population, such as Jiangsu, Shanghai, and Zhejiang, also tend to have higher income. These results highlight the importance of considering distributional impacts and different populations in strategies aiming to mitigate the detrimental effects of electricity generation.

The modeling tools used for this study have only recently been applied in China. The InMAP model has been widely used to estimate the health impacts of air pollution, especially those associated with power plant emissions in the United States.^50,52^ InMAP-China was recently developed to estimate the air quality and human health impacts of emissions across China.^34^ Although as a reduced-complexity model, InMAP does not include detailed representations of complex atmospheric chemistry and physics, the model’s prediction of PM_2.5_ concentrations in China has been shown to be similar to those obtained with a state-of-the-science comprehensive regional-scale air quality model (the Community Multiscale Air Quality (CMAQ) modeling system).^34^ Prior studies conducting power systems modeling for China have not applied a unit commitment and economic dispatch model to simulate operations across the entire country at hourly resolution for a full year. The GridPath model used here captures essential constraints in power system operations and maximizes overall benefits by including nearly all load zones in China.^29^

Under a pledge of carbon neutrality by 2060, China’s power sector is beginning the process of decarbonization. Through a unit commitment model with economic dispatch, the power plants with the highest emissions and adverse health impacts can be recognized, and high-priority targets for retirement can be identified. Additionally, economic dispatch may serve as a transitional strategy as clean energy infrastructure is developed. As compared to the equal-share approach, economic dispatch will reduce emissions and air pollution in many regions in China. Internalizing health damage costs into the dispatch of power plants would further benefit human health in most regions. However, it is notable that this transition can strongly influence the distribution of power sector impacts due to spatial variations in electricity generation and population. Future research should investigate the impacts of the power sector’s transition on different socio-economic populations. It is also important to integrate health impacts into future power system planning. Many studies have estimated substantial health benefits associated with the power sector’s decarbonization in China due to decreased use of fossil fuels. However, most treat air quality and public health benefits as ancillary benefits.^53^ Additional efforts are needed to co-optimize health benefits and greenhouse emission reductions, and greater attention should be paid to the distributional impacts of power systems planning to ensure a cleaner and more equitable transition.

## Supporting Information Available

A detailed description of the modeled power system, sensitivity analysis, and emissions and health impacts under different scenarios (PDF)
