# Supplementary material for: The Health and Climate Benefits of Economic Dispatch in China’s Power System
Source: Environ Sci Technol. 2023 Feb 9;57(7):2898–906. doi: 10.1021/acs.est.2c05663 (PMC9948285; doi:10.1021/acs.est.2c05663)
Supplement: Supplementary file 1 — es2c05663_si_001.pdf [file es2c05663_si_001.pdf]

The health and climate benefits of economic dispatch in China's power system

Qian Luo[1], Fernando Garcia-Menendez[1], Haozhe Yang[2], Ranjit Deshmukh[2], Gang He[3], Jiang Lin[4,5,*], and Jeremiah X. Johnson[1]

[1]Department of Civil, Construction, and Environmental Engineering, North Carolina State University, Raleigh, NC, 27695, USA
[2]Bren School of Environmental Science and Management, University of California at Santa Barbara, Santa Barbara, CA, 93117, USA
[3]Department of Technology and Society, Stony Brook University, Stony Brook, NY, 11794, USA
[4]Department of Electricity Market and Policy, Lawrence Berkeley National Laboratory, Berkeley, CA, 94720, USA
[5]Department of Agricultural and Resources Economics, University of California at Berkeley, Berkeley, CA 94720, USA
[*]J_Lin@lbl.gov

Summary

Seven figure and five tables are included in the supporting information (9 pages in total).

Description of the power system

4,803 unique units are included in the modeled power grid, including 2,997 coal generators, 1,092 hydropower plants, 275 wind power plants, 177 solar power plants, 44 nuclear power plants, and 218 natural gas power plants. Installed coal capacity accounts for 54% of the total installed capacity (Figure S1). To account for the high variability in coal generation efficiency, we include heat rates for coal generators at the unit level (Figures S2 and S3). As the installed capacity for natural gas is much lower than that of coal and the efficiency of gas-fired generators does not vary considerably across units within a facility, we use facility-level data for gas-fired power plants. Fuel costs are shown in Table S1. The capacity of transmission lines connecting load zones and electricity transmission losses are from He et al.[1] Transmission costs are from Abhyankar et al., with different costs associated with cross-regional and cross-provincial transmission.[2] Hourly wind and solar capacity factors are from Abhyankar et al.[3] and monthly average hydropower capacity factors are from He et al.[1] A summary of annual average wind and solar capacity factors is shown in Figure S4. Regional loads are from Abhyankar et al. (2022) and Figure S5 shows the annual load in each load zone. Table S2 includes location and gross domestic product (GDP) information for each load zone used in this study. GDP is for year 2020.[4] Operational parameters of coal- and natural gas-fired generators are shown in Table S3. Additionally, we assumed that 5% of the 8,760 hours in a year are needed for maintenance for both coal- and gas-fired generators.

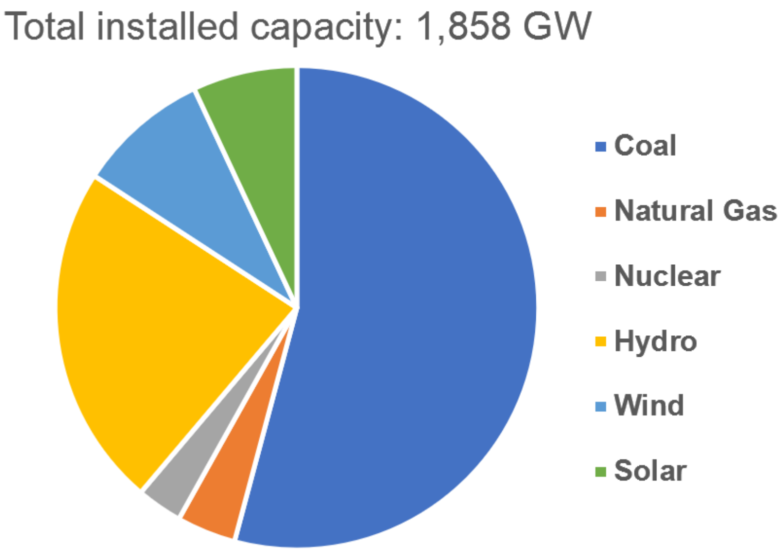

Figure S1. Installed generation capacity by fuel type in China in 2020.

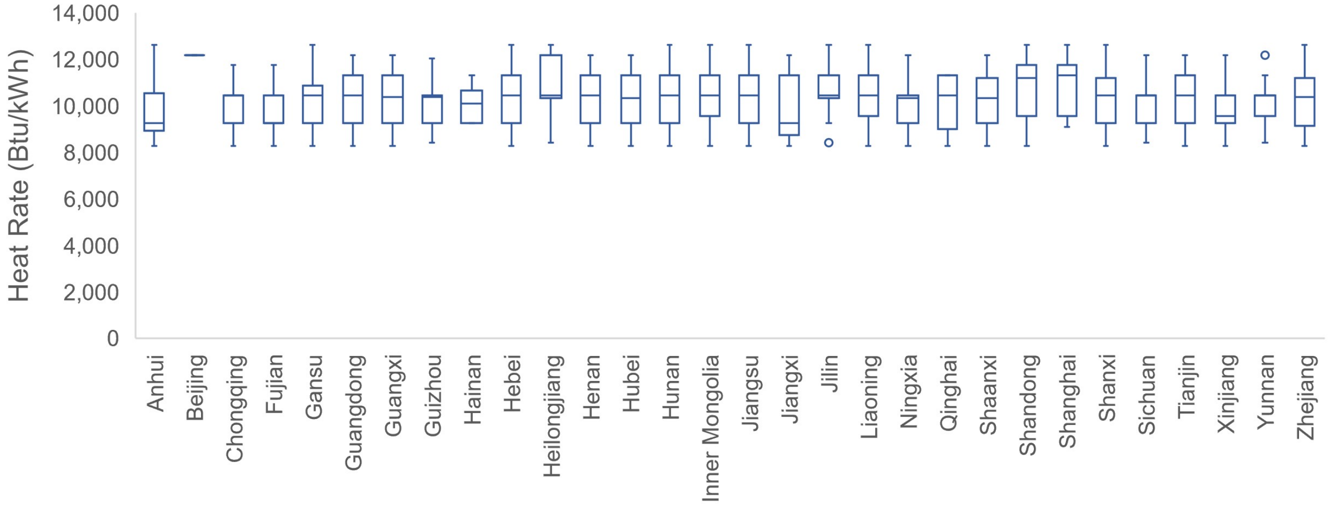

Figure S2. Boxplots showing distribution of unit-level heat rates of coal generators in each province in 2020. Box boundaries show interquartile range and lines indicate medians.

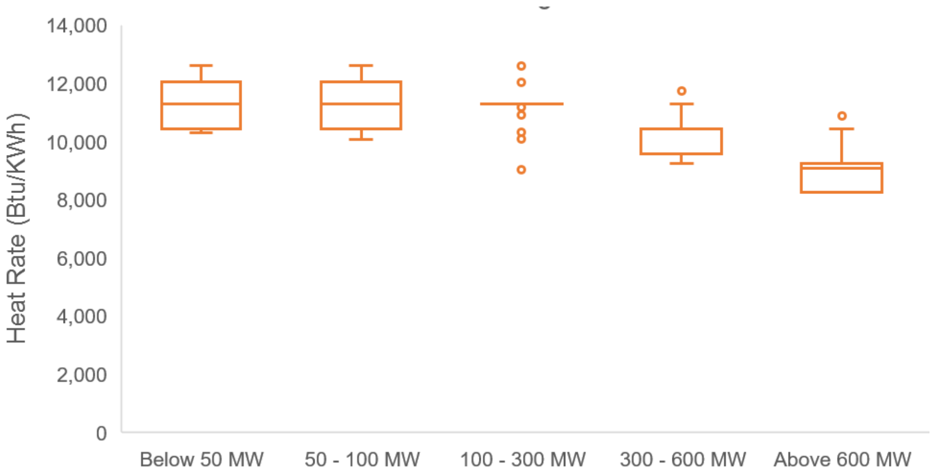

Figure S3. Boxplots showing distribution of unit-level heat rates of coal generators grouped by nameplate capacity. Box boundaries show interquartile range and lines indicate medians.

Table S1. Fuel prices used in the model. (He et al., 2020)

Fuel	Fuel Price ($/MMBtu)
Coal - Anhui	4.61
Coal - Beijing	4.93
Coal - Chongqing	3.83
Coal - East Inner Mongolia	2.80
Coal - Fujian	5.77
Coal - Gansu	3.60
Coal - Guangdong	6.17
Coal - Guangxi	6.17
Coal - Guizhou	6.17
Coal - Hebei	4.93
Coal - Heilongjiang	4.23
Coal - Henan	4.87
Coal - Hubei	4.41
Coal - Hunan	4.72
Coal - Jiangsu	5.77
Coal - Jiangxi	4.72
Coal - Jilin	3.15
Coal - Liaoning	3.38
Coal - Ningxia	3.19
Coal - Qinghai	3.09
Coal - Shaanxi	3.15
Coal - Shandong	4.93
Coal - Shanghai	5.77
Coal - Shanxi	4.22
Coal - Sichuan	3.82
Coal - Tianjin	4.93
Coal - West Inner Mongolia	2.80
Coal - Xinjiang	2.60
Coal - Yunnan	6.17
Coal - Zhejiang	5.77
Gas	13.68
Uranium	0.82

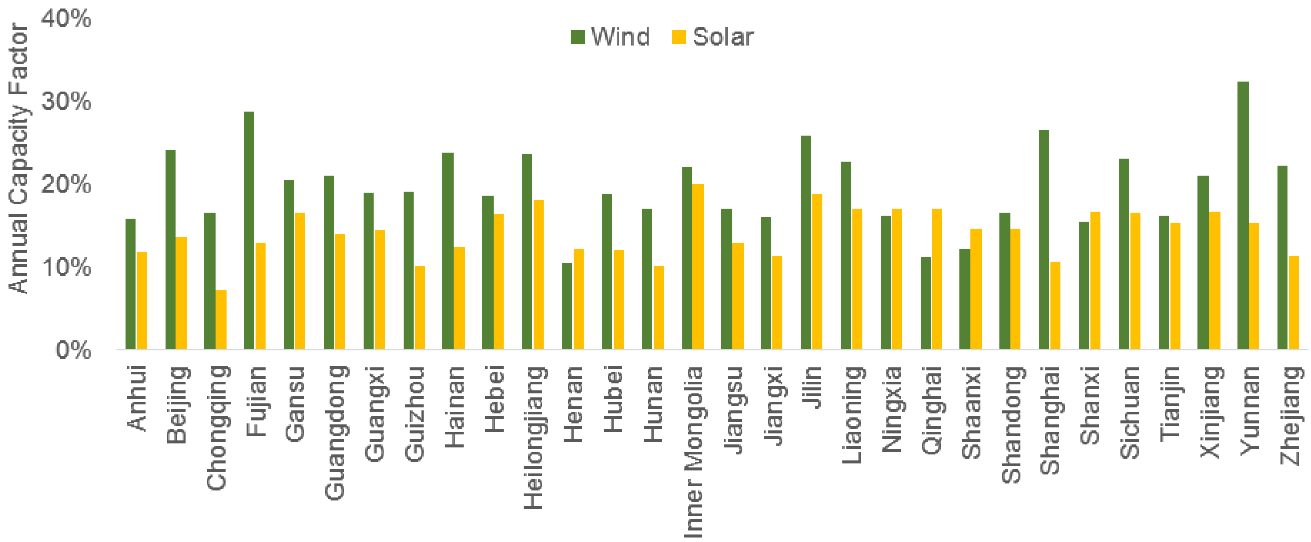

Figure S4. Annual average capacity factors for wind and solar energy in each province.

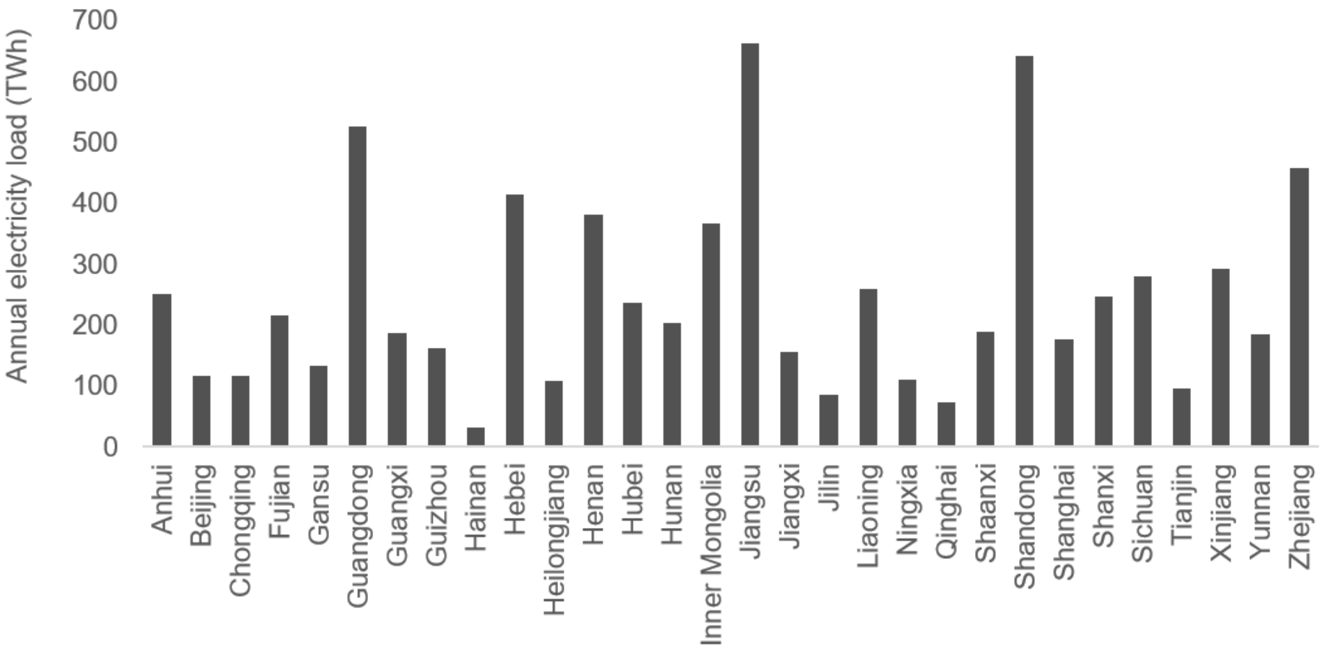

Figure S5. Total annual electricity demand in each province in 2020.

Table S2. Information for each load zone.

Full name	Abbreviation	Region	GDP per capita ($)
Guangdong	GD	Central South	12,743
Guangxi	GX	Central South	6,408
Hainan	HN	Central South	7,956
Henan	HEN	Central South	8,025
Hubei	HUB	Central South	10,906
Hunan	HUN	Central South	9,117
Anhui	AH	East	9,189
Fujian	FJ	East	15,323
Jiangsu	JS	East	17,573
Jiangxi	JX	East	8,243
Shangdong	SD	East	10,443
Shanghai	SH	East	22,560
Zhejiang	ZJ	East	14,508
Beijing	BJ	North	23,908
East Inner Mongolia	EIM	North	10,466
Hebei	HEB	North	7,036
Shanxi	SX	North	7,330
Tianjin	TJ	North	14,726
West Inner Mongolia	WIM	North	10,466
Heilongjiang	HLJ	Northeast	6,236
Jilin	JL	Northeast	7,414
Liaoning	LN	Northeast	8,649
Gansu	GS	Northwest	8,564
Ningxia	NX	Northwest	7,892
Qinghai	QH	Northwest	7,357
Shaanxi	SAX	Northwest	9,603
Xinjiang	XJ	Northwest	7,738
Chongqing	CQ	Southwest	11,309
Guizhou	GZ	Southwest	6,702
Sichuan	SC	Southwest	8,421
Yunnan	YN	Southwest	7,531

Table S3. Operational parameter assumptions for coal- and natural gas-fired generators.

Parameter	Unit	Coal	Gas
Ramp rate	% of capacity per hour	20	40
Minimum run time	Hours	12	6
Minimum down time	Hours	12	6

Sensitivity analysis

To test the model's responses to different transmission cost assumptions, we use three sets of transmission costs for electricity transported across provinces/regional power grids: $15/30 (used in our main analysis, from Abhyankar et al. (2020)), $10/20, $5/10, and $0/0 per MWh. The annual electricity generation change between equal-share and economic dispatch at the province level is shown in Figure S6. Coal-fired generation in each province is sensitive to whether and what transmission cost is included in the model. When no transmission costs are considered, electricity generation significantly increases in the northwest due to lower coal prices. However, when transmission costs rise to $10/20 or $15/30 per MWh, coal-fired generation increases in the east and drops in the northwest, as it is more expensive to transport cheaper electricity from the west to the east after considering transmission costs.

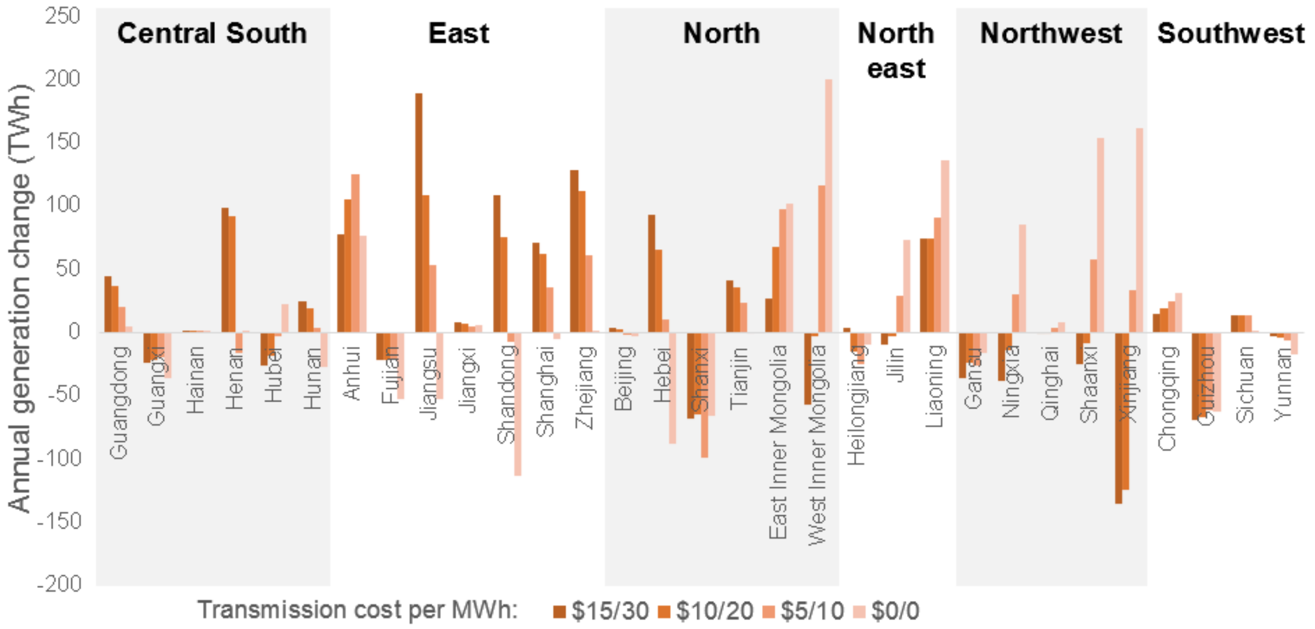

Figure S6. Coal-fired electricity generation change between equal-share dispatch and economic dispatch under different transmission cost assumptions ($/MWh across provinces and across regional power grids).

In addition to analyze the model's responses to different transmission costs, we aslo simulate electricity generation under economic dispatch without renewable energy curtailment. Compared with the results in the main analysis, coal generation in Gansu, Xinjiang, Qinghai, and Wes Inner Mongolia would be reduced by 3.3%, 3.0%, 1.7%, and 1.2%, respectively. Other regions are largely not affected by renewable energy curtailment. This change would not affect the overall distribution of electricity generation or health impacts. Our analysis, based on renewable profiles after curtailment, is a more conservative estimate of health and climate benefits from economic dispatch.

Power plant emissions and health impacts

We compare our emissions estimates under equal-share dispatch with two recent studies estimating air pollutant emissions from Chinese power plants in Table S4.[5,6] Our estimates are within the range of these studies, with values closer to those reported by Tang et al., which are based on a continuous emissions monitoring systems network. The most recent data from the two datasets are from 2017. China introduced ultra-low emission (ULE) standards in 2014 and most coal power plants achieved compliance with all ULE emission limits by the end of 2017.[7] We do not expect significant additional power sector emission changes from 2017 to 2020. Table S5 includes emissions and mortality changes relative to economic dispatch under emission externalities internalization scenarios. Figure S7 shows marginal emission costs for NO_x and $PM_{2.5}$ in each load zone.

Table S4. Total annual power sector emissions estimated in this study and estimates reported by prior studies.

	Annual total emissions (M tons)		
Pollutants	Tang et al	MEIC	This study
PM	0.20	1.70	0.24
SO_2	0.75	1.60	1.06
NO_x	1.25	4.50	1.37

Table S5. Reductions in emissions (CO_2, SO_2, NO_x, and $PM_{2.5}$) and premature mortality attributed to power plant emissions in China with climate and health damages internalization into power sector economic dispatch operations. Climate cost internalization scenarios are based on carbon prices of \$25/ton CO_2 (CP25) and \$55/ton CO_2 (CP55). Health damages internalization scenarios are based on regionally-varying (HD6cities) and national-level VSL estimates of \$1.2M (HD1M) and \$9.5M (HD9M). Reduced emissions, premature deaths, and percentages reported are relative to economic dispatch operations without climate and health damages internalization.

	CP25	CP55	HD1M	HD9M	HD6cities
CO_2 emitted (M tons per year)	71 (1.5%)	242 (5.2%)	245 (5.2%)	243 (5.2%)	144 (3.1%)
SO_2 emitted (Tons per year)	27,700 (3.3%)	82,600 (9.9%)	53,300 (6.4%)	132,000 (15.7%)	35,000 (4.2%)
NO_x emitted (Tons per year)	25,200 (2.1%)	45,500 (3.8%)	31,400 (2.6%)	20,900 (1.8%)	28,600 (2.4%)
$PM_{2.5}$ emitted (Tons per year)	23,600 (2.9%)	24,200 (5.1%)	24,600 (6.5%)	24,200 (5.2%)	24,600 (6.4%)
Premature deaths (per year)	2,560 (3.7%)	3,460 (5.1%)	8,340 (12.2%)	17,400 (25.4%)	4,590 (6.7)%)

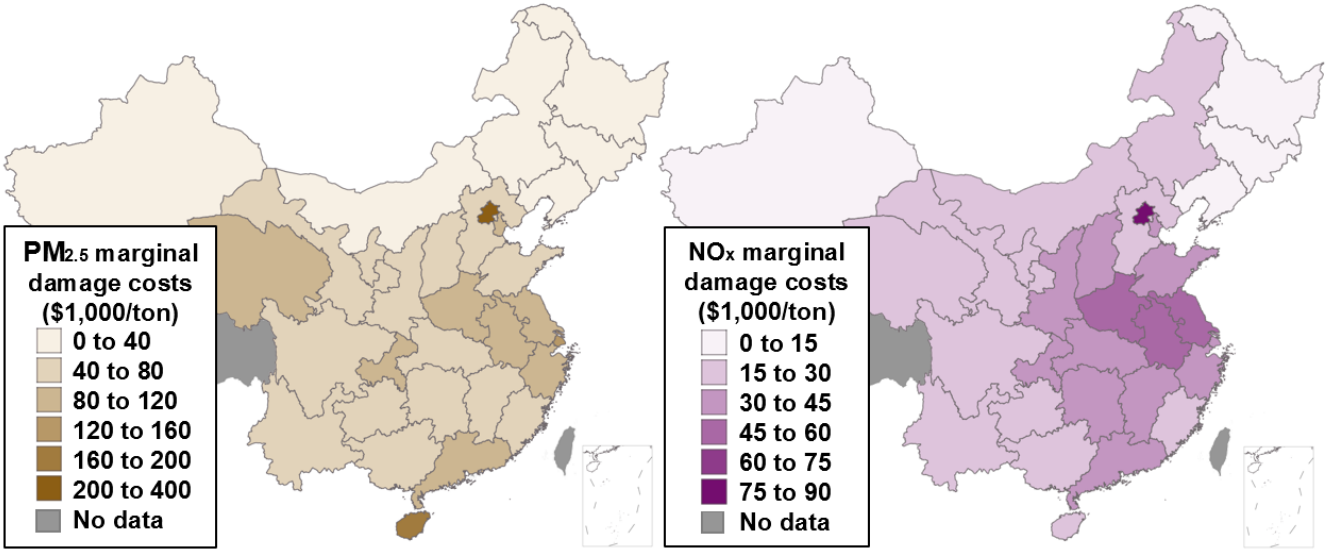

Figure S7. Province-level marginal damage costs from power plant NO_2 and $PM_{2.5}$ emissions in mainland China. Value of Statistic Life of \$1.2 million is used.

References

(1) He, G.; Lin, J.; Sifuentes, F.; Liu, X.; Abhyankar, N.; Phadke, A. Rapid cost decrease of renewables and storage accelerates the decarbonization of China's power system. *Nature communications* **2020**, *11*, 1–9.

(2) Abhyankar, N.; Lin, J.; Liu, X.; Sifuentes, F. Economic and environmental benefits of market-based power-system reform in China: A case study of the Southern grid system. *Resources, Conservation and Recycling* **2020**, *153*, 104558.

(3) Abhyankar, N.; Lin, J.; Kahrl, F.; Yin, S.; Paliwal, U.; Liu, X.; Khanna, N.; Luo, Q.; Wooley, D.; O'Boyle, M.; Ashmoore, O.; Orvis, R.; Solomon, M.; Phadke, A. Achieving an 80% carbon free electricity system in China by 2035. *iScience* **2022**, 105180.

(4) National Bureau of Statistics of China Per Capita Gross Regional Product in China, https://data.stats.gov.cn/english/easyquery.htm?cn=E0103, accessed 22 April 2022.

(5) Tang, L.; Xue, X.; Qu, J.; Mi, Z.; Bo, X.; Chang, X.; Wang, S.; Li, S.; Cui, W.; Dong, G. Air pollution emissions from Chinese power plants based on the continuous emission monitoring systems network. *Scientific Data* **2020**, *7*, 1–10.

(6) Tsinghua University Multi-resolution Emission Inventory for China (MEIC), http://meicmodel.org, (Accessed October 1, 2022).

(7) Tang, L.; Qu, J.; Mi, Z.; Bo, X.; Chang, X.; Anadon, L. D.; Wang, S.; Xue, X.; Li, S.; Wang, X.; Zhao, X. Substantial emission reductions from Chinese power plants after the introduction of ultra-low emissions standards. *Nature Energy* **2019**, *4*, 929–938.
